# NETosis in the Kidney, Liver, and Lung of Mice With Cecal Ligation and Puncture‐Induced Sepsis and the Ameliorating Effects of Adipose‐Derived Stem Cell Exosomes

**DOI:** 10.1002/kjm2.70273

**Published:** 2026-08-19

**Authors:** Shao‐Chun Wu, Tsu‐Hsiang Lu, Yi‐Chan Wu, Chia‐Wei Lin, Chia‐Wen Tsai, Ching‐Hua Hsieh

**Affiliations:** ^1^ Department of Anesthesiology Kaohsiung Chang Gung Memorial Hospital and Chang Gung University College of Medicine Kaohsiung Taiwan; ^2^ Department of Plastic Surgery Kaohsiung Chang Gung Memorial Hospital and Chang Gung University College of Medicine Kaohsiung Taiwan; ^3^ Doctoral Program of Clinical and Experimental Medicine, College of Medicine National Sun Yat‐sen University Kaohsiung Taiwan

**Keywords:** adipose‐derived stem cell exosomes, citrullinated histone H3, NETosis, neutrophil extracellular traps, sepsis

## Abstract

Sepsis‐induced organ failure involves dysregulated neutrophil responses, including neutrophil extracellular trap (NET) formation, via NETosis. Exosomes generated from adipose‐derived stem cells (ADSCs) have shown potential in sepsis treatment. This study characterized organ‐specific NETosis in the kidney, liver, and lungs using a murine cecal ligation and puncture (CLP) model and assessed the modulatory effects of ADSC exosomes. Male C57BL/6 mice were grouped as follows: control, CLP, and CLP with ADSC exosome therapy (*n* = 6 per group). Plasma cell‐free DNA (cfDNA) and tissue citrullinated histone H3 (CitH3) levels were measured at 16 h postoperatively. Confocal immunofluorescence was performed by co‐staining for CitH3 and Ly6G. Organ‐derived Ly6G‐enriched neutrophils were subjected to quantitative reverse transcription polymerase chain reaction for seven NETosis‐related genes: *CYBB*, *Padi4*, *H2ac20*, *H2bc21*, *Nlrc4*, *Nlrp3*, and *Ripk3*. CLP significantly elevated plasma cfDNA and lung CitH3 levels, whereas ADSC exosome treatment reduced both. Kidney and liver CitH3 levels remained unchanged across the groups. Confocal imaging confirmed active NETosis predominantly in the lung, with minimal signals in the kidneys and liver. In kidney and liver neutrophils, only *Nlrp3* and *Ripk3* were significantly upregulated by CLP. In the lungs, all seven genes were significantly elevated; ADSC exosomes significantly reduced *Padi4*, *H2ac20*, *Nlrp3*, and *Ripk3*. CLP‐induced NETosis was more pronounced in the lungs at the protein and transcriptional levels, where ADSC exosomes broadly suppressed NETotic pathways. NETosis reduction by ADSC exosomes was not observed in renal and hepatic neutrophils, indicating their possible protective role in sepsis within the pulmonary system.

## 1 Introduction

Sepsis is a life‐threatening organ dysfunction resulting from an aberrant host response to infection [1]. Sepsis results in approximately 11 million fatalities per year globally and remains one of the primary causes of mortality in intensive care units [2]. Neutrophils, the most abundant circulating leukocytes, are pivotal in early innate immune responses during sepsis. Their excessive activation contributes substantially to tissue damage through the release of reactive oxygen species (ROS), proteases, and neutrophil extracellular traps (NETs) [3, 4].

NETs are extracellular complexes comprising decondensed chromatin, histones, and granule proteins, including myeloperoxidase (MPO) and neutrophil elastase, which are released by NETosis, a cell death process [5]. Two main forms of NETosis are recognized: suicidal NETosis, a lytic process dependent on ROS generated by NADPH oxidase (NOX2), encoded by the cytochrome b‐245 beta chain gene (*CYBB*), and vital NETosis, a non‐lytic process that preserves neutrophil effector function [4]. Although NETs provide antimicrobial defense, their excessive or dysregulated formation during sepsis exacerbates immunothrombosis, endothelial damage, coagulopathy, and multiple organ dysfunction syndrome [6].

NETs are associated with the pathophysiology of sepsis‐related organ disorders, such as acute kidney injury (AKI), hepatic dysfunction, and acute lung injury (ALI)/acute respiratory distress syndrome (ARDS) [7, 8]. Patients with sepsis have higher circulating indicators of NET formation, including cell‐free DNA (cfDNA), MPO‐DNA complexes, and citrullinated histone H3 (CitH3), which are associated with organ failure and mortality [9, 10]. CitH3, generated by the enzymatic action of peptidylarginine deiminase 4 (PAD4, encoded by *Padi4*), is a well‐validated marker of NETosis, as histone citrullination promotes the chromatin decondensation required for NET release [11].

Beyond PAD4 and NOX2, emerging evidence has implicated the NLRP3 inflammasome and receptor‐interacting protein kinase 3 (RIPK3) in NETosis [12]. The NLRP3 inflammasome is assembled in neutrophils in a PAD4‐dependent manner and supports NETosis through nuclear envelope rupture mediated by caspase‐1 and gasdermin D [12]. RIPK3‐driven necroptosis is linked to NETosis, sharing downstream effectors of mixed‐lineage kinase domain‐like (MLKL) activation and membrane permeabilization [13]. Additionally, histone modifications, including histone H2A variant acetylation of H2ac20 (H2A clustered histone 20) and H2bc21 (H2B clustered histone 21), and NLRC4 inflammasome activation, participate in chromatin remodeling and neutrophil‐mediated inflammatory cascades during NETosis [14, 15].

Approximately 50% of patients with severe sepsis experience ALI or ARDS, with transmigration of neutrophils into the lungs being a critical pathophysiological event [16, 17]. Sepsis‐associated AKI occurs in approximately 60% of patients with sepsis, with NETs promoting glomerular microthrombi and tubular damage [18, 19]. In the liver, neutrophil infiltration and NETosis contribute to hepatic sinusoidal occlusion and hepatocellular injury [20]. However, the organ‐specific extent of NETosis at the molecular level and its modulation across these organs remain unclear.

Adipose‐derived stem cell (ADSC) exosomes are nanoscale extracellular vesicles that convey bioactive materials, including proteins, lipids, and non‐coding RNAs, and have been shown to be effective in mitigating sepsis‐induced lung injury [21, 22]. Wang et al. demonstrated that ADSC exosomes alleviated sepsis‐induced lung injury by suppressing interleukin‐27 secretion from macrophages in cecal ligation and puncture (CLP) mice [23]. Zou et al. reported that mesenchymal stem cell‐derived exosomes reduced excessive NET formation in septic lungs [24]. Furthermore, our group previously reported that in CLP mice, ADSC exosome treatment improved survival, reduced lung pathology, and modulated immune cell transcriptomes through multi‐cell‐type immunomodulation [25]. However, whether ADSC exosomes also attenuate NETosis in organs has not yet been systematically evaluated.

This study aimed to: (i) characterize the extent of NETosis across the kidney, liver, and lung in a septic CLP mouse model at 16 h postoperatively; (ii) assess circulating cfDNA and tissue CitH3 as markers of NET formation; (iii) visualize NETosis in situ by confocal immunofluorescence; and (iv) quantify mRNA expression of seven key NETosis‐related genes (*CYBB*, *Padi4*, *H2ac20*, *H2bc21*, *Nlrc4*, *Nlrp3*, and *Ripk3*) in the lymphocyte antigen 6 complex, locus G6D (Ly6G)‐enriched neutrophils isolated from each organ following CLP with or without ADSC exosome treatment.

## 2 Materials and Methods

### 2.1 ADSC Culture and Exosome Purification

Mouse ADSCs (catalog no. 100MU006, iXCells Biotechnologies, San Diego, CA, USA) were cultured in ADSC growth medium (catalog no. DM‐0003, iXCells Biotechnologies) supplemented with 100 units/mL of penicillin‐streptomycin (Gibco, Thermo Fisher Scientific) and maintained at 37°C in a humidified atmosphere containing 5% CO_2_. To ensure experimental reproducibility and cellular homogeneity, cells at passages 3–5 were used exclusively. Prior to experimental use, ADSC identity and purity were authenticated by multicolor flow cytometric analysis, confirming the robust expression of mesenchymal stromal cell‐associated surface markers, including CD29, CD44, CD73, CD90, and CD105, along with the absence of hematopoietic lineage markers CD34 and CD45; this is consistent with the established criteria for mesenchymal stem cell characterization.

For exosome isolation, ADSCs were seeded and cultured until they reached approximately 80% confluence, at which point the standard growth medium was replaced with serum‐free ADSC basal medium to eliminate potential contamination from bovine‐derived extracellular vesicles. The conditioned medium was harvested after 48 h of serum‐free culture, a timeframe optimized to maximize the exosome yield while maintaining cell viability. The conditioned medium was subjected to sequential differential centrifugation, first at 300*g* for 10 min to pellet and remove intact cells, followed by a second centrifugation step at 3000*g* for 15 min to eliminate residual cell debris and apoptotic bodies. A clear supernatant was subsequently passed through a 0.22 μm polyethersulfone membrane filter to remove the remaining microvesicles and particulate contaminants, thereby enriching the preparation for small extracellular vesicles within the exosomal size range.

Exosome precipitation was performed using ExoQuick‐TC solution (catalog no. EXOTC50A‐1, System Biosciences, Palo Alto, CA, USA) according to the manufacturer's recommended protocol. Briefly, the filtered conditioned medium was combined with ExoQuick‐TC at a volumetric ratio of 5:1, thoroughly mixed by inversion, and incubated overnight at 4°C to facilitate polymer‐based precipitation of extracellular vesicles. Following incubation, the mixture was centrifuged at 1500*g* for 30 min at 4°C, and the resulting exosome‐enriched pellet was carefully decanted and resuspended in an appropriate volume of sterile phosphate‐buffered saline (PBS, pH 7.4) for downstream analysis and functional assays. Total protein concentration of the exosome preparations was quantified using a bicinchoninic acid (BCA) protein assay kit, with bovine serum albumin as the standard curve reference.

Comprehensive physicochemical and biochemical characterization of the isolated exosomes was subsequently performed using complementary approaches. Morphological integrity and vesicle ultrastructure were assessed using transmission electron microscopy, which confirmed a characteristic cup‐shaped or spherical bilayered membranous morphology. Particle size distribution and concentration were determined by nanoparticle tracking analysis, demonstrating a predominant size range of 50–150 nm, which is consistent with the accepted biophysical parameters of exosomes. Molecular identity was further validated by western blotting for the exosomal tetraspanin markers CD9, CD81, Alix, the endosomal sorting complex required for transport‐associated protein TSG101, and the negative control protein calnexin (Figure S1), collectively confirming successful isolation of authentic exosomes as previously described [26, 27, 28, 29].

### 2.2 Animals and Sepsis Model

Male C57BL/6 mice (8–10 weeks old, 25–30 g; National Laboratory Animal Center, NARLabs, Taiwan) were housed under specific pathogen‐free conditions with a 12‐h light/dark cycle at 22°C ± 2°C, with ad libitum access to standard chow and water. All experimental procedures were approved by the Institutional Animal Care and Use Committee and conducted in accordance with the Guide for the Care and Use of Laboratory Animals.

The mice were randomly assigned to three groups (*n* = 6 per group): (i) control (CTL) group, which is a sham‐operated control group in which the cecum was surgically exposed, but neither ligated nor punctured; (ii) CLP group, in which polymicrobial sepsis was induced by CLP; and (iii) CLP + EXO group, in which CLP‐operated mice received therapeutic ADSC exosomes. Sepsis was established using a well‐validated CLP model as previously described [25, 30]. Under isoflurane anesthesia, the cecum was ligated below the ileocecal valve, punctured once with a 21‐gauge needle, and gently compressed to extrude a small amount of fecal material into the peritoneal cavity. Following cecal closure, all CLP mice received subcutaneous saline resuscitation at a dose of 5 mL/100 g body weight.

Mice in the CLP + EXO group received two intravenous injections of 100 μg ADSC exosomes via the tail vein at 4‐h intervals following surgery, yielding a cumulative dose of 200 μg per animal. The exosome dosing regimen was adopted from our previously published CLP study [25], in which the same two‐dose intravenous protocol (100 μg at 0 and 4 h post‐CLP, making a total of 200 μg per animal) was validated and shown to improve survival, attenuate lung pathology, and modulate immune cell transcriptomes without observable adverse effects. All mice were sacrificed 16 h postoperatively, and biochemical, histological, and molecular analyses were performed using peripheral blood and target organs.

### 2.3 Plasma cfDNA Quantification

Peripheral blood samples were collected via cardiac puncture into ethylenediaminetetraacetic acid‐coated tubes to prevent coagulation and minimize ex vivo DNA release. Plasma was obtained by centrifugation at 2000*g* for 10 min at 4°C, and the clear supernatant was carefully aspirated and transferred into clean microcentrifuge tubes, thereby avoiding disturbance of the buffy coat layer and preventing cellular contamination of the plasma fraction. The samples were processed promptly or stored at −80°C until analysis.

Plasma cfDNA concentration was quantified using a Qubit 1× dsDNA high‐sensitivity assay kit (catalog no. Q32851; Invitrogen/Thermo Fisher Scientific, Waltham, MA, USA), which is a fluorescence‐based assay optimized for accurate detection of low‐abundance double‐stranded DNA in complex biological matrices. Quantification was performed according to the manufacturer's recommended protocol using a Qubit Fluorometer with a standard curve established from the provided DNA standards. cfDNA concentrations were calculated from fluorescence readings and expressed in picograms per milliliter (pg/mL) of plasma, reflecting the systemic burden of cell‐free nucleic acids as a marker of cellular injury and inflammatory activity.

### 2.4 Tissue CitH3 Enzyme‐Linked Immunosorbent Assay (ELISA)

The kidney, liver, and lung tissues were harvested at the time of sacrifice and immediately snap‐frozen in liquid nitrogen to preserve protein integrity. The tissues were subsequently homogenized in ice‐cold radioimmunoprecipitation assay buffer supplemented with a protease inhibitor cocktail to prevent protein degradation during extraction. Homogenates were centrifuged at 12,000*g* for 15 min at 4°C, and the clear supernatants were collected for downstream analysis. To ensure equal loading across samples, the total protein concentration of each homogenate was determined using the BCA assay.

CitH3, a well‐established molecular marker of NET formation, was quantified in tissue homogenates using a CitH3 (Clone 11D3) ELISA kit (catalog no. 501620; Cayman Chemical, Ann Arbor, MI, USA) according to the manufacturer's instructions. Briefly, the samples and standards were incubated in antibody‐coated microplate wells, followed by sequential incubation with detection antibodies and enzyme substrate solutions. Absorbance was measured at 450 nm using a microplate reader, and the CitH3 concentrations were interpolated from a standard curve. Results were expressed as pg/mL of tissue homogenate, reflecting the degree of NET‐mediated tissue involvement across the assessed organ compartments.

### 2.5 Confocal Immunofluorescence

The tissue samples were fixed in 4% paraformaldehyde at 4°C overnight, cryoprotected in a sucrose gradient, and embedded in an optimal cutting temperature compound. Immunofluorescence staining was performed on 5 μm tissue sections from the kidney, liver, and lung to assess NET formation. Following deparaffinization, rehydration, antigen retrieval, and blocking, the sections were incubated overnight at 4°C with rabbit anti‐mouse CitH3 (Catalog No. ab5103, Abcam) and rat anti‐mouse Ly6G (catalog no. 31469, Cell Signaling Technology) primary antibodies. The sections were subsequently incubated with Alexa Fluor 488 goat anti‐rabbit (catalog no. ab150077, Abcam) and Alexa Fluor 594 goat anti‐rat (catalog no. 405422, BioLegend) secondary antibodies. The nuclei were counterstained with 4′,6‐diamidino‐2‐phenylindole (DAPI). Rat IgG2a and rabbit IgG served as isotype controls. Slides were imaged by confocal microscopy, and co‐localization of CitH3 and Ly6G signals was interpreted as indicative of active NET formation.

### 2.6 Neutrophil Isolation From Tissues

The kidneys, livers, and lungs were rinsed twice with PBS and incubated with Accutase Solution (catalog no. A6964, Sigma‐Aldrich) at 37°C for 15 min in 10 mL volume on culture dishes. Tissue fragments were mechanically dissociated with a syringe plunger and filtered through a 70‐μm cell strainer on ice, and all centrifugation steps used precooled buffers to minimize ex vivo activation. The cell suspensions were layered onto Ficoll (2:1 tissue:Ficoll ratio, 400*g* for 30 min), and the buffy coat was collected. After two washes in Dulbecco's PBS (250*g*, 7 min), neutrophils were positively selected using anti‐mouse Ly‐6G and Ly‐6C particles‐DM (BD IMag, catalog no. 558111, BD Biosciences) according to the manufacturer's protocol, including rotational incubation at 6°C–12°C for 15 min, followed by three magnetic separations using BD IMag buffer. Flow cytometric purity assessment was performed using anti‐Ly6G antibody (clone 1A8) at the time of isolation, and the mean purity of Ly6G‐positive cells across preparations was 85% ± 6% (mean ± standard deviation). The purified neutrophils were preserved in an RNA stabilization solution (RNAprotect Tissue Reagent, catalog no. 76104, QIAGEN) and stored at −80°C.

### 2.7 RNA Extraction and Quantitative Reverse Transcription Polymerase Chain Reaction (RT‐qPCR)

Total RNA was extracted from tissue samples using a miRNeasy Mini kit (catalog no. 217004; QIAGEN, Hilden, Germany) according to the manufacturer's instructions, and RNA integrity and concentration were assessed by spectrophotometry. Complementary DNA (cDNA) was synthesized from 100 ng of total RNA using a High‐Capacity cDNA Reverse Transcription kit (catalog no. 4368814; Applied Biosystems, Foster City, CA, USA). RT‐qPCR was subsequently performed using Power SYBR Green PCR Master Mix (catalog no. 4367659; Applied Biosystems) on an ABI StepOnePlus Real‐Time PCR system. Relative gene expression was calculated using the 2^−ΔΔ*C*^_t_ method with *GAPDH* as the internal reference gene. The following primers were used:

*CYBB*: Forward 5′‐CGTTGAGTGGTGTGTGAATGCC‐3′, reverse 5′‐TGTGATCCCAGCCAACCGAG‐3′.

*Padi4*: Forward 5′‐GCTATCAGCTGTTCCAGGAGCTAC‐3′, reverse 5′‐TCCAGTCGATACAGCTCTCCACAT‐3′.

*H2ac20*: Forward 5′‐CATCATCCCGCGCCATCTG‐3′, reverse 5′‐CTTGCTCTTAGCCTTGTGGCTCTC‐3′.

*H2bc21*: Forward 5′‐AAGGGTTCCAAGAAGGCTGT‐3′, reverse 5′‐ACAAACGAGTTCATGATGCCCA‐3′.

*Nlrc4*: Forward 5′‐ACGAAGCCTGAAGAAGATGCGT‐3′, reverse 5′‐TTTCACAGAGTTTGCAGTCAGACAGC‐3′.

*Nlrp3*: Forward 5′‐AGCTCTGACCTCTGTGCTCAAA‐3′, reverse 5′‐GCTGCAGTTGTCTAATTCCAGCATC‐3′.

*Ripk3*: Forward 5′‐CGTCCTCCACTGACAGAGC‐3′, reverse 5′‐TTACCTCGGAGACAGCAGCAT‐3′.

*GAPDH*: Forward 5′‐GCACAGTCAAGGCCGAGAAT‐3′, reverse 5′‐GCCTTCTCCATGGTGGTG‐3′.

### 2.8 Statistical Analysis

All data are presented as means ± standard error of the mean. Group comparisons were performed using one‐way analysis of variance, followed by the Tukey's honest significant difference post hoc test to identify pairwise differences while controlling for multiple comparisons. Statistical significance was set at *p* < 0.05. All statistical analyses were conducted using IBM SPSS Statistics software (version 26.0; IBM Corp., Armonk, NY, USA). All the experiments were performed in at least three independent replicates, and outliers were identified and assessed using Grubbs' test prior to statistical analysis.

## 3 Results

### 3.1 CLP Increases Plasma cfDNA and Lung CitH3 With Reduction by ADSC Exosome Treatment

**FIGURE 1:**
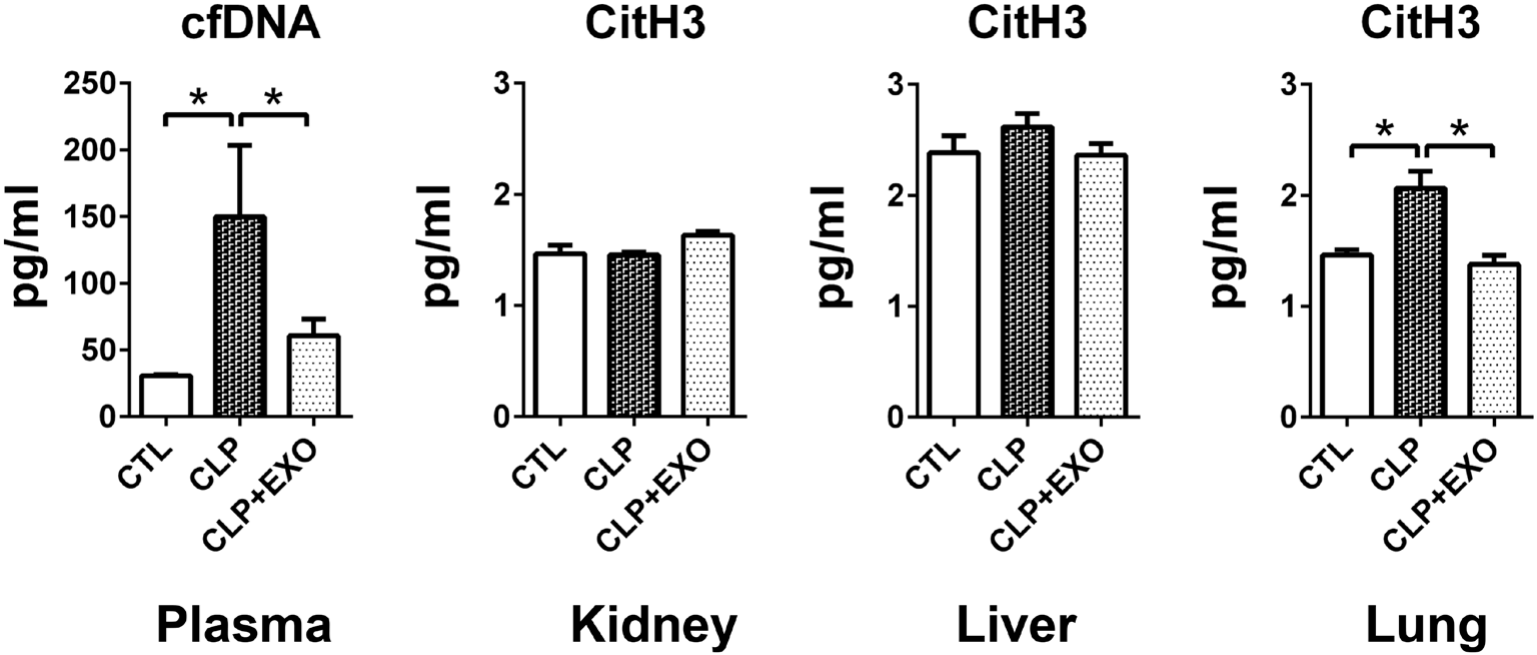
Systemic and tissue‐specific biomarkers of NETosis. Effect of adipose‐derived stem cell (ADSC) exosomes on systemic and organ‐specific neutrophil extracellular trap (NET) biomarkers in septic mice. Plasma cell‐free DNA (cfDNA) levels were significantly elevated at 16 h of post‐cecal ligation and puncture (CLP) compared with those in control (CTL) animals, a response that was significantly attenuated by treatment with ADSC exosomes (CLP + EXO). Within the tissues, citrullinated histone H3 (CitH3) levels, measured by enzyme‐linked immunosorbent assay (ELISA), showed a significant increase in the lungs following CLP, which was similarly reduced by exosome administration. In contrast, CitH3 concentrations in kidney and liver homogenates did not exhibit significant differences among the three experimental groups at this time point. Statistical data are presented as means ± standard error of the mean (SEM) with *n* = 6 mice per group, where *p < 0.05 denotes significance between the indicated groups.

As shown in Figure 1, plasma cfDNA was significantly elevated in the CLP group compared with the CTL group (150 vs. 33 pg/mL, *p* < 0.05), consistent with increased systemic NET‐associated DNA release during sepsis. Treatment with ADSC exosomes significantly reduced plasma cfDNA levels to 60 pg/mL (*p* < 0.05 vs. CLP), suggesting that exosome therapy suppressed systemic NET formation or facilitated NET clearance.

CitH3 in the lungs was significantly elevated in the CLP group compared with that in the CTL group (2.1 vs. 1.5 pg/mL, *p* < 0.05), and ADSC exosome treatment significantly reduced lung CitH3 to 1.4 pg/mL (*p* < 0.05 vs. CLP). This pattern indicates that NETosis was particularly prominent in the lungs during CLP‐induced sepsis. In contrast, neither the kidney nor liver CitH3 differed significantly across the three groups (1.4–1.5 pg/mL for the kidney and 2.4–2.7 pg/mL for the liver), suggesting that citrullination‐dependent NET formation in these organs was not significantly altered by CLP or exosome treatment at the 16‐h time point assessed.

### 3.2 Confocal Immunofluorescence Reveals Sparse NETosis Signals in the Kidney and Liver but Increased NET Formation in the Lung

**FIGURE 2:**
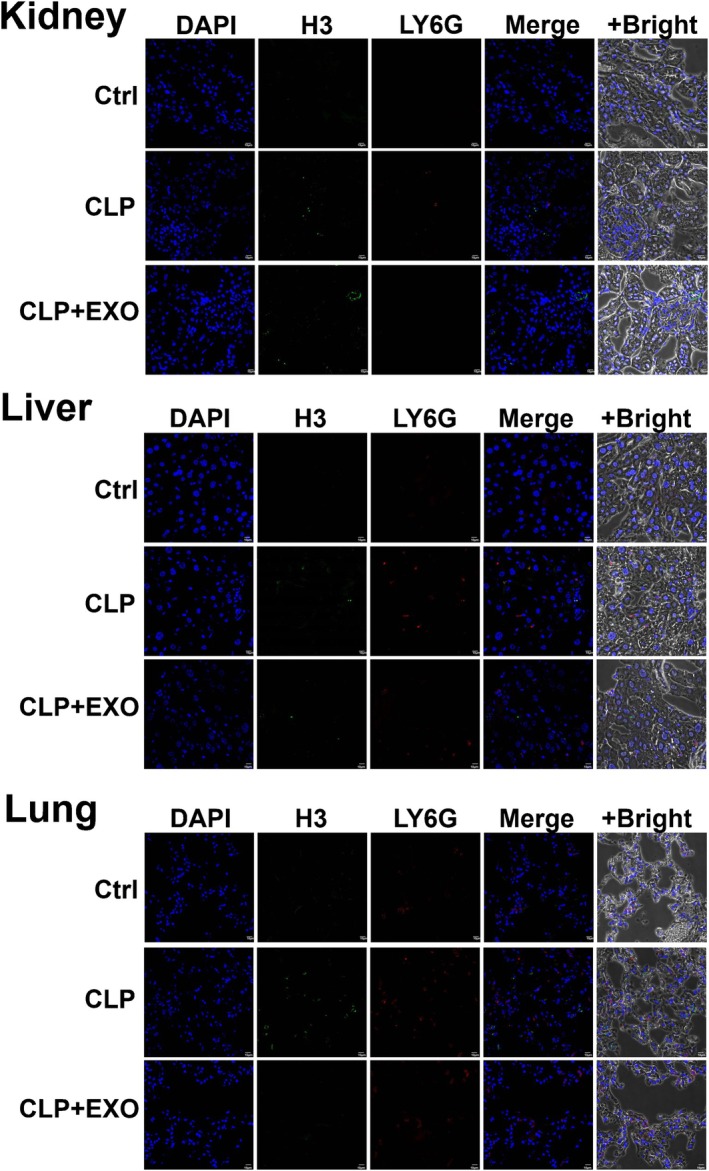
Visual characterization of NETosis in situ. Representative confocal immunofluorescence images showing neutrophil extracellular traps (NETs) in different organs. Tissue sections from the kidney, liver, and lung were stained with DAPI for nuclei (blue), anti‐histone H3 (citrullinated histone H3 [CitH3]) to identify NETosis (green), and anti‐Ly6G to mark neutrophils (red). In the lungs, double‐positive signals for CitH3 and Ly6G were markedly increased in the cecal ligation and puncture (CLP) group relative to those in the control (CTL) group, indicating active NET formation in situ, whereas exosome treatment reduced these signals. Conversely, the kidney and liver exhibited sparse and comparable NETosis signals across all the three groups, suggesting limited NET formation in these compartments at 16 h after sepsis induction.

Confocal immunofluorescence using DAPI, anti‐histone H3 (CitH3), and anti‐Ly6G antibodies was performed on tissue sections from all three organs (Figure 2). In the kidney, CitH3‐ and Ly6G‐positive signals were sparse and comparable across the CTL, CLP, and CLP + EXO groups, which was consistent with the lack of significant differences in tissue CitH3 levels measured by ELISA. Similarly, in the liver, the co‐localization of CitH3 and Ly6G signals was minimal across all groups. These findings suggest that NETosis at the tissue level in the kidney and liver was limited or below the detection threshold of immunofluorescence at the 16‐h endpoint.

In the lungs, CitH3 and Ly6G double‐positive signals, which represented neutrophils undergoing NETosis in situ, were more apparent in the CLP group than in the CTL group. Exosome treatment in the CLP + EXO group reduced these signals, which was consistent with the ELISA results. Taken together, the lungs appeared to be the primary site for active NETosis, as evidenced by both the biochemical and microscopic approaches.

### 3.3 NETosis‐Related Gene Expression in Isolated Neutrophils Is Organ‐Specific

**FIGURE 3:**
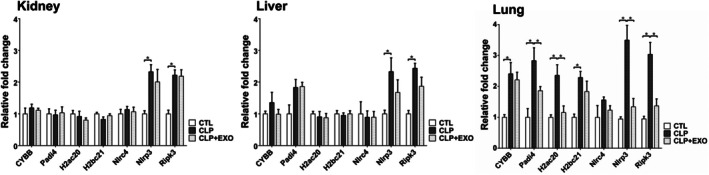
Transcriptional profiles of isolated neutrophils. The organ‐specific mRNA expression profiles of seven key NETosis‐related genes (*CYBB*, *Padi4*, *H2ac20*, *H2bc21*, *Nlrc4*, *Nlrp3*, and *Ripk3*) in Ly6G‐enriched neutrophils isolated from the kidney, liver, and lung. In the kidney and liver, sepsis induction via cecal ligation and puncture (CLP) led to a targeted upregulation of Nlrp3 and Ripk3 without significantly altering other markers. However, the lungs showed broad transcriptional activation, with all the seven genes significantly upregulated in the CLP group. Adipose‐derived stem cell (ADSC) exosome treatment comprehensively modulated the lung response by significantly reducing the expression of *Padi4*, *H2ac20*, *Nlrp3*, and *Ripk3*. Relative mRNA expression was calculated using the 2^−ΔΔ*C*^_T_ method and normalized to glyceraldehyde 3‐phosphate dehydrogenase, with statistical significance indicated by *p < 0.05.

To characterize NETosis at the molecular level, Ly6G‐enriched neutrophils were isolated from the kidney, liver, and lung tissues, and the expression of seven NETosis‐related genes was quantified using RT‐qPCR (Figure 3).

#### 3.3.1 Kidney

In the kidney, *Nlrp3* and *Ripk3* expression were both significantly upregulated in the CLP group compared with those in the CTL group (2.2‐fold and 2.1‐fold, respectively; *p* < 0.05), whereas *CYBB*, *Padi4*, *H2ac20*, *H2bc21*, and *Nlrc4* did not differ significantly across the groups. ADSC exosome treatment did not significantly reduce *Nlrp3* or *Ripk3* expression compared with CLP alone, although both tended to be lower. These results suggest that in the kidney, NETosis‐associated gene induction during sepsis is primarily driven by the NLRP3 and RIPK3 pathways rather than the canonical PAD4–ROS axis.

#### 3.3.2 Liver

In the liver, *Nlrp3* and *Ripk3* expression were significantly elevated in the CLP group compared with those in the CTL group (2.3‐fold and 2.4‐fold, respectively; *p* < 0.05), recapitulating the pattern observed in the kidney. The remaining genes (*CYBB*, *Padi4*, *H2ac20*, *H2bc21*, and *Nlrc4*) in the liver were not significantly altered by CLP. Exosome treatment did not produce a statistically significant reduction in *Nlrp3* or *Ripk3*, indicating that the therapeutic effect of exosomes on hepatic NETosis‐associated gene expression was limited at this time point.

#### 3.3.3 Lung

The lungs exhibited the most extensive upregulation of NETosis‐related genes. In the CLP group, *CYBB* (2.4‐fold), *Padi4* (2.8‐fold), *H2ac20* (2.5‐fold), *H2bc21* (2.2‐fold), *Nlrc4* (1.9‐fold), *Nlrp3* (3.3‐fold), and *Ripk3* (3.0‐fold) levels were significantly elevated compared with those in the CTL group (*p* < 0.05). Treatment with ADSC exosomes significantly reduced *Padi4*, *H2ac20*, *Nlrp3*, and *Ripk3* expression (*p* < 0.05 vs. CLP), while *CYBB*, *H2bc21*, and *Nlrc4* expression also decreased. These data indicate that ADSC exosomes broadly attenuate NETosis‐related gene activation in lung neutrophils during sepsis, encompassing both the PAD4‐dependent citrullination and NLRP3/RIPK3‐driven cell death pathways.

## 4 Discussion

This study demonstrated organ‐specific patterns of NETosis in CLP‐induced murine sepsis and identified ADSC exosomes as agents that broadly attenuate NETosis‐related gene expression in the lungs, while partially moderating it in the kidneys and liver. The principal findings were as follows: (i) plasma cfDNA and lung CitH3 were significantly elevated in the CLP mice and reduced by ADSC exosome treatment; (ii) confocal imaging localized active NETosis predominantly to the lung; (iii) *Nlrp3* and *Ripk3* were consistently upregulated in the kidney, liver, and lung neutrophils; and (iv) the lungs showed the broader transcriptional NETosis signature encompassing *CYBB, Padi4, H2ac20, H2bc21*, and *Nlrc4* in addition to *Nlrp3* and *Ripk3*, which was more comprehensively suppressed by ADSC exosome treatment.

The elevation of plasma cfDNA in the CLP mice was consistent with established evidence that sepsis triggers NET‐associated DNA release into circulation [9]. In a systematic review and meta‐analysis, Charoensappakit et al. established that cfDNA functions as both a diagnostic and prognostic biomarker for adult sepsis [31]. cfDNA reduction by ADSC exosomes aligned with the reports by Zou et al., which showed that mesenchymal stem cell‐derived exosomes suppressed NET formation and reduced circulating dsDNA in CLP mice [24]. Furthermore, this finding is consistent with our previous single‐cell RNA‐sequencing study, in which ADSC exosomes downregulated pro‐inflammatory and oxidative burst pathways in lung neutrophils [25].

The predominance of NETosis in the lungs relative to the kidney and liver, as evidenced by elevated CitH3 levels detected by ELISA and immunofluorescence, reflects the well‐established susceptibility of the lungs to sepsis‐associated neutrophil infiltration and NET deposition. In patients with sepsis developing ALI or ARDS, neutrophil transmigration and activation in the lungs are early, central events [3]. Consistent with our ELISA findings, Alsabani et al. reported that targeting CXCR1/CXCR2 signaling reduced NETosis, thrombosis, and lung injury in CLP mice [6]. The relatively limited CitH3 signal in the kidney and liver at 16 h may reflect the time course of organ‐specific neutrophil accumulation rather than the true absence of NETosis, as renal and hepatic neutrophil infiltration may peak at different time points than that in the lungs [7, 20]. Time‐course validation is necessary to obtain definitive conclusions regarding organ‐specific NETosis routes.

The selective upregulation of *Nlrp3* and *Ripk3* in kidney and liver neutrophils without significant changes in *CYBB* or *Padi4* suggests that in these organs, alternative non‐canonical NETosis pathways, which are driven by the NLRP3 inflammasome and RIPK3‐mediated necroptosis, may predominate over the classic NOX2–ROS–PAD4 axis at this time point. Karmakar et al. supported this interpretation by demonstrating that NLRP3 inflammasome assembly in neutrophils is PAD4‐dependent and promotes NETosis under sterile circumstances [32]. RIPK3 and its downstream effector MLKL regulate necroptotic cell death, which overlaps mechanistically with NETosis through shared membrane‐permeabilizing events [33]. The fact that *Nlrp3* and *Ripk3* are upregulated without PAD4 elevation in the kidney and liver may indicate an inflammasome‐ and necroptosis‐driven NETosis route that does not require extensive citrullination of histones in these organs, which is consistent with the absence of significantly elevated CitH3 levels by ELISA in the kidney and liver.

In the lungs, the comprehensive upregulation of all seven tested NETosis‐related genes supports the notion that both the canonical (NOX2–PAD4) and non‐canonical (NLRP3, RIPK3) pathways are simultaneously engaged during septic lung injury. *CYBB* encodes NOX2, the catalytic subunit of NADPH oxidase, which is essential for ROS‐dependent suicidal NETosis [4, 5]. *Padi4* drives histone citrullination, enabling chromatin decondensation necessary for NET extrusion [11]. Upregulation of *H2ac20* and *H2bc21* reflects histone remodeling during NETosis, as modifications of histone H2A and H2B variants facilitate chromatin loosening [14]. *Nlrc4* encodes the NLR family CARD domain‐containing protein 4, an alternative inflammasome component that interacts with the NLRP3 pathway and promotes neutrophil‐mediated inflammation [15]. The simultaneous involvement of these molecular nodes highlights NETosis complexity in septic lungs.

ADSC exosome treatment significantly suppressed multiple NETosis‐related genes in the lungs. The significant reduction in *Padi4*, *H2ac20*, *Nlrp3*, and *Ripk3* expression following exosome treatment was consistent with the immunomodulatory effect that simultaneously targeted PAD4, H2ac20, NLRP3, and RIPK3. This multi‐nodal suppression may explain why ADSC exosomes reduce both cfDNA and tissue CitH3 levels, as NETs arising from each pathway contribute to extracellular DNA and citrullinated histone release. The mechanism by which ADSC exosomes exert these effects likely involves the transfer of miRNAs, proteins, and lipids that modulate intracellular signaling in recipient neutrophils [34]. For example, Shen et al. identified circular RNA circ‐Fryl in ADSC exosomes that promoted pro‐survival autophagy via the miR‐490‐3p/SIRT3 axis, blocking cell death pathways that culminate in NETosis [35]. Lu et al. reported that mesenchymal stem cell‐derived extracellular vesicles transferred mitochondria to modulate NET formation during ischemia–reperfusion injury [36]. Collectively, exosomal cargo may simultaneously dampen ROS generation, limit NLRP3 inflammasome priming, and reduce RIPK3‐dependent necroptosis, resulting in comprehensive suppression of NETs in the lungs.

In the kidney and liver, ADSC exosome treatment did not significantly reduce *Nlrp3* or *Ripk3* expression, despite its positive effects in the lung. This discrepancy may reflect the relatively lower baseline NETosis burden in these organs at 16 h, the organ‐specific bioavailability of intravenously administered exosomes, or the distinct kinetics of neutrophil infiltration in each organ. The CLP model produces time‐dependent organ injury patterns, and it is possible that kidney and liver NETosis peaks at later time points not captured here [37]. Future studies should examine the time‐course changes at 24 and 48 h in all three organs to determine whether exosome efficacy differs across organs or time points.

The limited effect of ADSC exosomes on kidney and liver NETosis‐related gene expression also raises the question of whether targeted delivery, for example, using organ‐homing exosome formulations, could enhance efficacy in these compartments. Sharma et al. noted that low yield, targeting efficiency, and variability in exosomal cargo represent translational challenges that must be overcome before clinical application [38]. Standardizing exosome production to ensure consistent anti‐NETosis cargo and investigating organ‐specific delivery strategies will be important in the future.

This study has several limitations. First, the sample size of six mice per group was relatively small; larger studies are needed to confirm organ‐specific differences. Second, only a single time point (16 h) was examined; thus, a longitudinal assessment would provide more complete information on the kinetics of NETosis. Third, elevated Nlrp3 and Ripk3 mRNA levels in the kidney and liver neutrophils were indicative of transcriptional priming of non‐canonical NETosis pathways. However, without protein‐level corroboration, it was insufficient to conclude whether these pathways were functionally active. Future validation of the protein levels of NLRP3, RIPK3, and PAD4 in isolated neutrophils may help elucidate the involved pathways. Fourth, this study used only male mice. Therefore, sex‐based differences in neutrophil responses during sepsis should be explored [37]. Fifth, as discussed by Wu et al. [25], we acknowledge the inherent limitations of the CLP model in recapitulating human sepsis, particularly the immunological complexity and heterogeneity of clinical sepsis. Finally, functional studies involving genetic knockdown or pharmacological inhibition of *Nlrp3* or *Ripk3* would provide causal evidence for the observed changes in gene expression.

## 5 Conclusion

CLP‐induced sepsis produces organ‐specific NETosis, most prominently in the lungs, where both the canonical PAD4–NOX2 and non‐canonical NLRP3–RIPK3 pathways are simultaneously activated. In contrast, kidney and liver neutrophils exhibit selective upregulation of *Nlrp3* and *Ripk3* without significant citrullinated histone elevation. ADSC exosomes significantly reduced NETosis at both the protein and transcriptional levels in the lungs, but not in renal and hepatic neutrophils, indicating their possible protective role in sepsis within the pulmonary system.

## Funding

This work was supported by Chang Gung Memorial Hospital (CMRPG8P0341).

## Conflicts of Interest

The authors declare no conflicts of interest.

## Supporting information


**Figure S1:** Western blotting analysis to confirm exosome characterization.

## Data Availability

The data that support the findings of this study are available from the corresponding author upon reasonable request.

## References

[1] M. Singer , C. S. Deutschman , C. W. Seymour , et al., “The Third International Consensus Definitions for Sepsis and Septic Shock (Sepsis‐3),” JAMA 315 (2016): 801–810. 26903338 10.1001/jama.2016.0287 PMC4968574

[2] K. E. Rudd , S. C. Johnson , K. M. Agesa , et al., “Global, Regional, and National Sepsis Incidence and Mortality, 1990‐2017: Analysis for the Global Burden of Disease Study,” Lancet 395 (2020): 200–211. 31954465 10.1016/S0140-6736(19)32989-7 PMC6970225

[3] J. Grommes and O. Soehnlein , “Contribution of Neutrophils to Acute Lung Injury,” Molecular Medicine 17 (2011): 293–307. 21046059 10.2119/molmed.2010.00138 PMC3060975

[4] P. Xu , Z. Tao , and C. Zhang , “Integrated Multi‐Omics and Artificial Intelligence to Explore New Neutrophils Clusters and Potential Biomarkers in Sepsis With Experimental Validation,” Frontiers in Immunology 15 (2024): 1377817. 38868781 10.3389/fimmu.2024.1377817 PMC11167131

[5] V. Brinkmann , U. Reichard , C. Goosmann , et al., “Neutrophil Extracellular Traps Kill Bacteria,” Science 303 (2004): 1532–1535. 15001782 10.1126/science.1092385

[6] M. Alsabani , S. T. Abrams , Z. Cheng , et al., “Reduction of NETosis by Targeting CXCR1/2 Reduces Thrombosis, Lung Injury, and Mortality in Experimental Human and Murine Sepsis,” British Journal of Anaesthesia 128 (2022): 283–293. 34893315 10.1016/j.bja.2021.10.039 PMC8792833

[7] J. He , F. Zheng , L. Qiu , et al., “Plasma Neutrophil Extracellular Traps in Patients With Sepsis‐Induced Acute Kidney Injury Serve as a New Biomarker to Predict 28‐Day Survival Outcomes of Disease,” Frontiers in Medicine 11 (2024): 1496966. 39629231 10.3389/fmed.2024.1496966 PMC11611547

[8] L. Morimont , M. Dechamps , C. David , et al., “NETosis and Nucleosome Biomarkers in Septic Shock and Critical COVID‐19 Patients: An Observational Study,” Biomolecules 12 (2022): 1038. 36008932 10.3390/biom12081038 PMC9405965

[9] M. Haem Rahimi , F. Bidar , A. C. Lukaszewicz , et al., “Association of Pronounced Elevation of NET Formation and Nucleosome Biomarkers With Mortality in Patients With Septic Shock,” Annals of Intensive Care 13 (2023): 102. 37847336 10.1186/s13613-023-01204-y PMC10581968

[10] D. Zhang , J. Guo , C. Shi , et al., “MPO‐DNA Complexes and cf‐DNA in Patients With Sepsis and Their Clinical Value,” Biomedicine 12 (2024): 2190. 10.3390/biomedicines12102190 PMC11505433 39457503

[11] S. Masuda , D. Nakazawa , H. Shida , et al., “NETosis Markers: Quest for Specific, Objective, and Quantitative Markers,” Clinica Chimica Acta 459 (2016): 89–93. 10.1016/j.cca.2016.05.029 27259468

[12] P. Münzer , R. Negro , S. Fukui , et al., “NLRP3 Inflammasome Assembly in Neutrophils Is Supported by PAD4 and Promotes NETosis Under Sterile Conditions,” Frontiers in Immunology 12 (2021): 683803. 34122445 10.3389/fimmu.2021.683803 PMC8195330

[13] A. A. D'Cruz , M. Speir , M. Bliss‐Moreau , et al., “The Pseudokinase MLKL Activates PAD4‐Dependent NET Formation in Necroptotic Neutrophils,” Science Signaling 11 (2018): eaao1716. 30181240 10.1126/scisignal.aao1716 PMC6301070

[14] J. Huang , W. Hong , M. Wan , and L. Zheng , “Molecular Mechanisms and Therapeutic Target of NETosis in Diseases,” MedComm 3 (2022): e162. 36000086 10.1002/mco2.162 PMC9390875

[15] A. Malik and T. D. Kanneganti , “Inflammasome Activation and Assembly at a Glance,” Journal of Cell Science 130 (2017): 3955–3963. 29196474 10.1242/jcs.207365 PMC5769591

[16] J. E. Sevransky , M. M. Levy , and J. J. Marini , “Mechanical Ventilation in Sepsis‐Induced Acute Lung Injury/Acute Respiratory Distress Syndrome: An Evidence‐Based Review,” Critical Care Medicine 32 (2004): S548–S553. 15542963 10.1097/01.ccm.0000145947.19077.25

[17] X. Xu , Q. Zhang , Z. Lv , et al., “Unraveling the Deadly Dance: Endothelial Cells and Neutrophils in Sepsis‐Induced Acute Lung Injury/Acute Respiratory Distress Syndrome,” Frontiers in Cell and Developmental Biology 13 (2025): 1551138. 40476000 10.3389/fcell.2025.1551138 PMC12137324

[18] D. Nakazawa , J. A. Marschner , L. Platen , and H. J. Anders , “Extracellular Traps in Kidney Disease,” Kidney International 94 (2018): 1087–1098. 30466565 10.1016/j.kint.2018.08.035

[19] N. Lerolle , D. Nochy , E. Guérot , et al., “Histopathology of Septic Shock Induced Acute Kidney Injury: Apoptosis and Leukocytic Infiltration,” Intensive Care Medicine 36 (2010): 471–478. 19924395 10.1007/s00134-009-1723-x

[20] F. Wang , M. Chen , J. Ma , et al., “Integrating Bulk and Single‐Cell Sequencing Reveals the Phenotype‐Associated Cell Subpopulations in Sepsis‐Induced Acute Lung Injury,” Frontiers in Immunology 13 (2022): 981784. 36405762 10.3389/fimmu.2022.981784 PMC9666384

[21] P. Hong , H. Yang , Y. Wu , K. Li , and Z. Tang , “The Functions and Clinical Application Potential of Exosomes Derived From Adipose Mesenchymal Stem Cells: A Comprehensive Review,” Stem Cell Research & Therapy 10 (2019): 242. 31391108 10.1186/s13287-019-1358-y PMC6686455

[22] K. S. Papadopoulos , C. Piperi , and P. Korkolopoulou , “Clinical Applications of Adipose‐Derived Stem Cell (ADSC) Exosomes in Tissue Regeneration,” International Journal of Molecular Sciences 25 (2024): 5916. 38892103 10.3390/ijms25115916 PMC11172884

[23] X. Wang , D. Liu , X. Zhang , L. Yang , Z. Xia , and Q. Zhang , “Exosomes From Adipose‐Derived Mesenchymal Stem Cells Alleviate Sepsis‐Induced Lung Injury in Mice by Inhibiting the Secretion of IL‐27 in Macrophages,” Cell Death Discovery 8 (2022): 18. 35013123 10.1038/s41420-021-00785-6 PMC8744023

[24] T. Zou , J. Lu , Y. Zhu , Y. Xu , and Y. Sun , “Mesenchymal Stem Cell‐Derived Exosomes Improved Septic Lung Injury by Reducing Excessive NETs Formation and Alleviating Inflammatory Response,” Allergologia et Immunopathologia 53 (2025): 63–68. 39786877 10.15586/aei.v53i1.1238

[25] S. C. Wu , C. S. Rau , Y. C. Wu , et al., “Single‐Cell Transcriptomic Analysis Reveals Therapeutic Mechanisms of Adipose‐Derived Stem Cell Exosomes in Sepsis‐Induced Lung Injury,” International Journal of Surgery 111 (2025): 9049–9064. 40607951 10.1097/JS9.0000000000002894 PMC12695251

[26] C. S. Rau , S. C. Wu , P. J. Kuo , et al., “Tracking Adipose‐Derived Stem Cell Exosomes Applied in a Mouse Crush Injury Model: Insights From Fluorescent Labeling and Spatial Transcriptomics—An Experimental Study,” International Journal of Surgery 111 (2025): 1860–1873. 39705130 10.1097/JS9.0000000000002166

[27] S. C. Wu , P. J. Kuo , C. S. Rau , et al., “Increased Angiogenesis by Exosomes Secreted by Adipose‐Derived Stem Cells Upon Lipopolysaccharide Stimulation,” International Journal of Molecular Sciences 22 (2021): 8877. 34445582 10.3390/ijms22168877 PMC8396299

[28] P. J. Kuo , C. S. Rau , S. C. Wu , et al., “Exosomes Secreted by Adipose‐Derived Stem Cells Following FK506 Stimulation Reduce Autophagy of Macrophages in Spine After Nerve Crush Injury,” International Journal of Molecular Sciences 22 (2021): 9628. 34502537 10.3390/ijms22179628 PMC8431814

[29] S. C. Wu , C. S. Rau , Y. C. Wu , et al., “Circulating Exosomes From Septic Mice Activate NF‐κB/MIR17HG Pathway in Macrophages,” Biomedicine 12 (2024): 534. 10.3390/biomedicines12030534 PMC10968321 38540147

[30] M. G. Toscano , D. Ganea , and A. M. Gamero , “Cecal Ligation Puncture Procedure,” Journal of Visualized Experiments 7 (2011): 2860. 10.3791/2860 PMC3339843 21587163

[31] A. Charoensappakit , K. Sae‐khow , N. Vutthikraivit , et al., “Immune Suppressive Activities of Low‐Density Neutrophils in Sepsis and Potential Use as a Novel Biomarker of Sepsis‐Induced Immune Suppression,” Scientific Reports 15 (2025): 9458. 40108283 10.1038/s41598-025-92417-7 PMC11923122

[32] M. Karmakar , M. Minns , E. N. Greenberg , et al., “N‐GSDMD Trafficking to Neutrophil Organelles Facilitates IL‐1β Release Independently of Plasma Membrane Pores and Pyroptosis,” Nature Communications 11 (2020): 2212. 10.1038/s41467-020-16043-9 PMC7200749 32371889

[33] S. Ciftel , A. Klisic , E. Ciftel , et al., “Investigating the Hepatic Response to Orlistat and White Tea in Rats on a High‐Fat Diet,” Life 14 (2024): 1283. 39459583 10.3390/life14101283 PMC11509274

[34] D. Shen and Z. He , “Mesenchymal Stem Cell‐Derived Exosomes Regulate the Polarization and Inflammatory Response of Macrophages via miR‐21‐5p to Promote Repair After Myocardial Reperfusion Injury,” Annals of Translational Medicine 9 (2021): 1323. 34532460 10.21037/atm-21-3557 PMC8422151

[35] W. Shen , X. Zhao , and S. Li , “Exosomes Derived From ADSCs Attenuate Sepsis‐Induced Lung Injury by Delivery of Circ‐Fryl and Regulation of the miR‐490‐3p/SIRT3 Pathway,” Inflammation 45 (2022): 331–342. 34478012 10.1007/s10753-021-01548-2

[36] T. Lu , J. Zhang , J. Cai , et al., “Extracellular Vesicles Derived From Mesenchymal Stromal Cells as Nanotherapeutics for Liver Ischaemia‐Reperfusion Injury by Transferring Mitochondria to Modulate the Formation of Neutrophil Extracellular Traps,” Biomaterials 284 (2022): 121486. 35447404 10.1016/j.biomaterials.2022.121486

[37] N. Wang , Y. Lu , J. Zheng , and X. Liu , “Of Mice and Men: Laboratory Murine Models for Recapitulating the Immunosuppression of Human Sepsis,” Frontiers in Immunology 13 (2022): 956448. 35990662 10.3389/fimmu.2022.956448 PMC9388785

[38] A. Sharma , A. Yadav , A. Nandy , and S. Ghatak , “Insight Into the Functional Dynamics and Challenges of Exosomes in Pharmaceutical Innovation and Precision Medicine,” Pharmaceutics 16 (2024): 709. 38931833 10.3390/pharmaceutics16060709 PMC11206934

